# Supplementary material for: Improved human greenspace exposure equality during 21st century urbanization
Source: Nat Commun. 2023 Oct 13;14:6460. doi: 10.1038/s41467-023-41620-z (PMC10575899; doi:10.1038/s41467-023-41620-z)
Supplement: Supplementary file 1 — Supplementary Information [file 41467_2023_41620_MOESM1_ESM.pdf]

Supporting Information for

Improved human greenspace exposure equality during 21st century urbanization

Shengbiao Wu[1], Bin Chen[1,2,3,*], Chris Webster[2,3,4], Bing Xu[5], and Peng Gong[2,6,7]

1. Future Urbanity & Sustainable Environment (FUSE) Lab, Division of Landscape Architecture, Department of Architecture, Faculty of Architecture, The University of Hong Kong, Hong Kong SAR, China
2. Urban Systems Institute, The University of Hong Kong, Hong Kong SAR, China
3. HKU Musketeers Foundation Institute of Data Science, The University of Hong Kong, Hong Kong SAR, China
4. HKUrbanLabs, Faculty of Architecture, The University of Hong Kong, Hong Kong SAR, China
5. Department of Earth System Science, Ministry of Education Ecological Field Station for East Asian Migratory Birds, and Institute for Global Change Studies, Tsinghua University, Beijing, 100084, China
6. Department of Geography, and Department of Earth Sciences, The University of Hong Kong, Hong Kong SAR, China
7. Institute for Climate and Carbon Neutrality, The University of Hong Kong, Hong Kong SAR, China

*Corresponding author: Bin Chen (binley.chen@hku.hk)

1. Supplementary information

1.1. Calculation of inequality index

In this study, we used three types of widely used measures[1-2] to quantify inequality in human exposure to greenspace, including Lorenz curve-based (i.e., Gini coefficient index), social welfare-based (i.e., Atkinson index), and generalized entropy metrics (i.e., Theil index). To consider the impacts of nearby green environments on the inequality measures, we conducted a buffer analysis on greenspace coverage with a size of 500 m using the image convolution algorithm with the "convolve" function in Google Earth Engine.

1.1.1. Gini index

As shown in **Supplementary Fig. 18**, the Gini index is calculated by the Lorenz curve framework, and defined as the ratio of the area that lies between the line of equality (i.e., straight diagonal line) and the Lorenz curve (i.e., cumulative share of greenspace exposure ranked by residents that are exposed from lowest to highest greenspace; region A) over the total area under the line of equality (region *A* plus region *B*):

$$Gini = \frac{s_A}{s_A+s_B} \qquad \text{(S1)}$$

where s_A and s_B represent the areas of regions *A* and *B*, respectively.

According to previous studies[3-4], Gini index can be mathematically formulated as:

$$Gini = 1 - \frac{\sum_{i=1}^{N}\sum_{j=1}^{i-1} g_j + \sum_{i=1}^{N}\sum_{j=1}^{i} g_j}{N \times \sum_{j=1}^{N} g_j} \qquad \text{(S2)}$$

where g_j is the greenspace coverage that exposed to j^{th} resident with a buffer size of *d* (500 m) and *N* is the number of total residents within the target city.

1.1.2. Atkinson index

The Atkinson index is another inequality metric based on the concept of welfare equivalent equally distributed greenspace, which is defined as the percentage of total greenspace for one society that must forego to have more equal shares of greenspace among individuals in that society[2]. The advantage of the Atkinson index is that it can provide a complete ranking of greenspace distribution in the explicitly social welfare function. Mathematically, the Atkinson index can be calculated as:

$$Atkinson\ (\varepsilon) = 1 - \left(\frac{1}{N}\sum_{j=1}^{N}\left(\frac{g_j}{\bar{g}}\right)^{1-\varepsilon}\right)^{\frac{1}{1-\varepsilon}} \qquad \varepsilon \neq 1 \qquad \text{(S3)}$$

$$Atkinson\ (\varepsilon) = 1 - \frac{\prod_{j=1}^{N}\left(g_j^{\frac{1}{N}}\right)}{\bar{g}} \qquad \varepsilon = 1 \qquad \text{(S4)}$$

where $\bar{g}$ represents the average greenspace exposure and ε is the inequality aversion parameter that regulates the sensitivity of the social welfare losses from inequality to greenspace exposure inequality. As ε increases, the Atkinson index is more sensitive to changes in the lower end of the greenspace distribution and vice versa.

1.1.3. Theil index

The Theil index is another generalized entropy indicator that measures the ranking greenspace inequality that can overcome the limitation of the Gini index when the Lorenz curves of the two target cities cross [2]. It is defined as:

$$Theil = \frac{1}{N}\sum_{j=1}^{N}\frac{g_j}{\bar{g}}ln\left(\frac{g_j}{\bar{g}}\right) \qquad \text{(S5)}$$

Supplementary figures

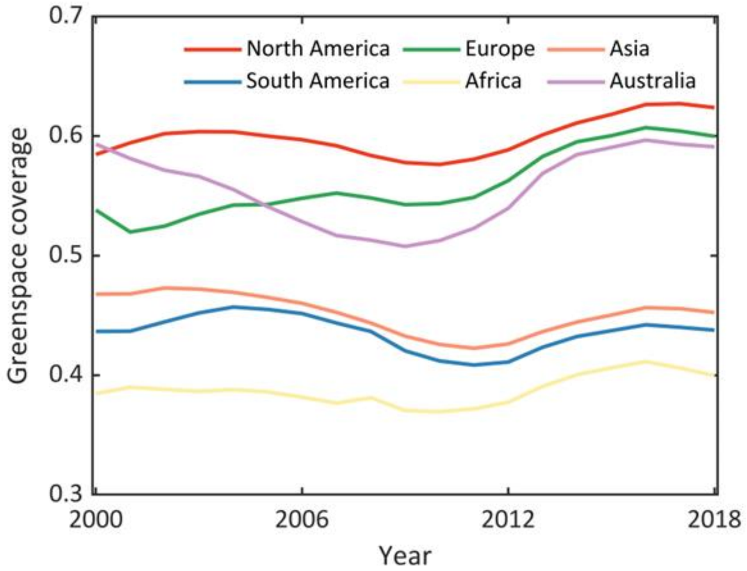

Supplementary Fig. 1. Continental-scale mean annual dynamics of physical greenspace coverage (GC) for global 1028 cities from 2000-2018.

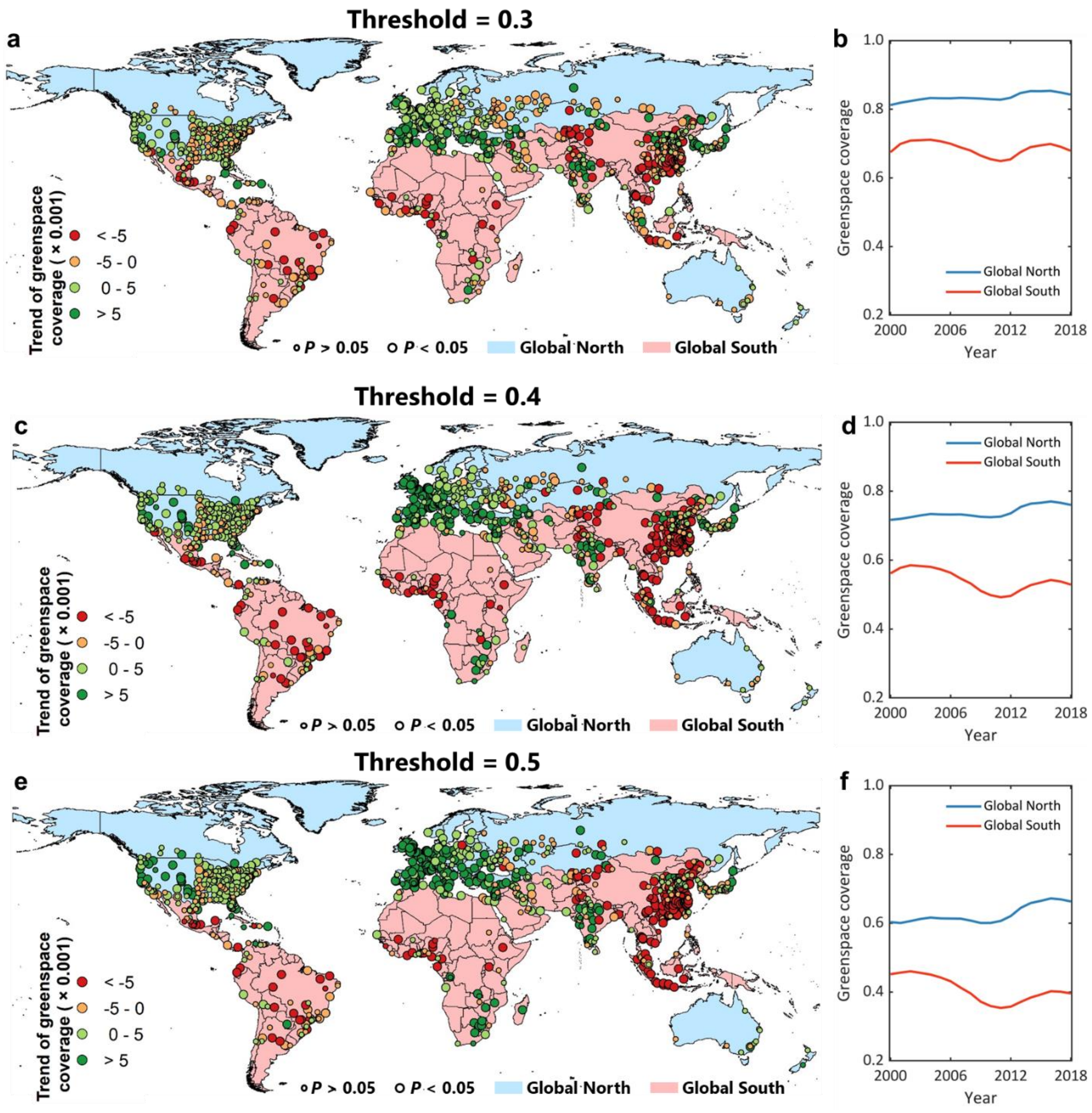

Supplementary Fig. 2. Sensitivity of threshold in the linear unmixing-based greenspace classification to the temporal changes of physical greenspace coverage (GC) for global 1028 cities from 2000-2018. a and **b.** Threshold = 0.3. **c** and **d.** Threshold = 0.4. **e** and **f.** Threshold = 0.5. First column is the city-level temporal trend of GC changes (**a, c,** and **e**). Second column is the mean annual GC dynamics for Global North and Global South cities (**b, d,** and **f**). The non-parametric Theil–Sen slope estimator approach is used to determine the long-term trends of GC. The non-parametric Mann-Kendall is used to evaluate the significance of these detected temporal trends. Large bubble sizes represent a statistically significant level of 0.05 (p-value <0.05) and small bubble sizes represent a non-significant trend with p-value >0.05. The administrative boundaries data is from the Global Administrative Areas (GADM) (https://gadm.org/).

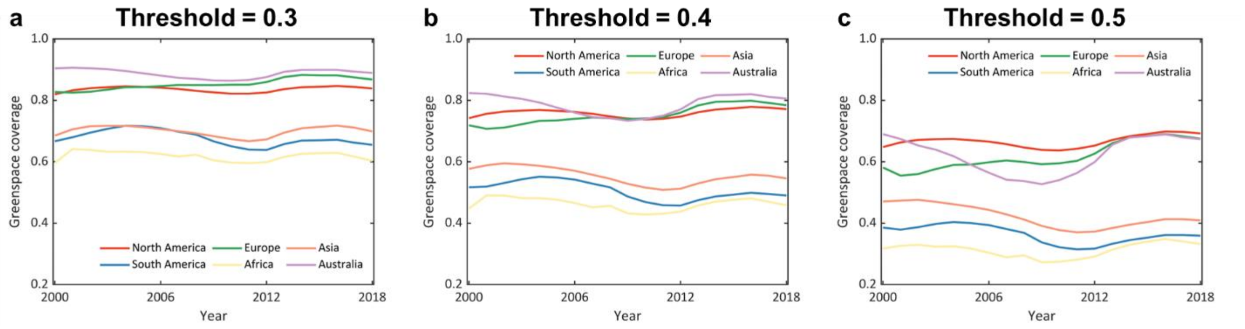

Supplementary Fig. 3. Sensitivity of threshold in the linear unmixing-based greenspace classification to the mean annual dynamics of physical greenspace coverage (GC) for global 1028 cities from 2000-2018. **a.** Threshold = 0.3. **b.** Threshold = 0.4. **c.** Threshold = 0.5.

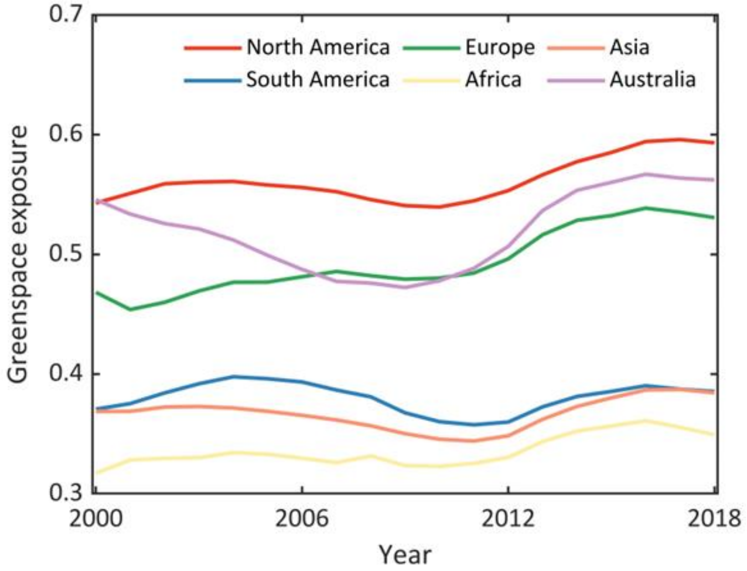

Supplementary Fig. 4. Continental-scale mean annual dynamics of real greenspace exposure (GE) for global 1028 cities from 2000-2018.

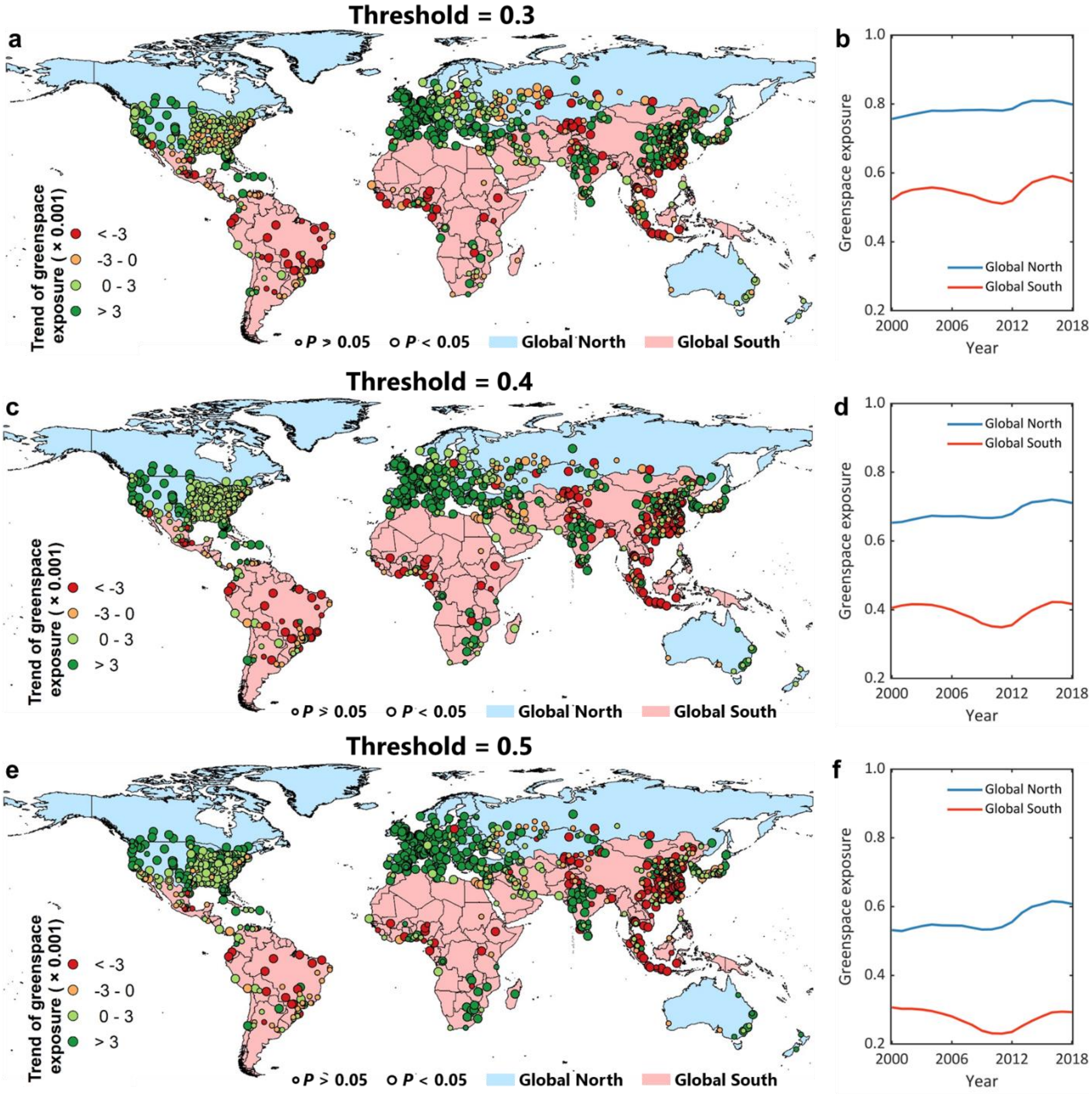

Supplementary Fig. 5. Sensitivity of threshold in the linear unmixing-based greenspace classification to the temporal changes of real greenspace exposure (GE) for global 1028 cities from 2000-2018. a and **b.** Threshold = 0.3. **c** and **d.** Threshold = 0.4. **e** and **f.** Threshold = 0.5. First column is the city-level temporal trend of GE changes (**a, c,** and **e**). Second column is the mean annual GE dynamics for Global North and Global South cities (**b, d,** and **f**). The non-parametric Theil–Sen slope estimator approach is used to determine the long-term trends of GE. The non-parametric Mann-Kendall is used to evaluate the significance of these detected temporal trends. Large bubble sizes represent a statistically significant level of 0.05 (*p*-value <0.05) and small bubble sizes represent a non-significant trend with *p*-value >0.05. The administrative boundaries data is from the Global Administrative Areas (GADM) (https://gadm.org/).

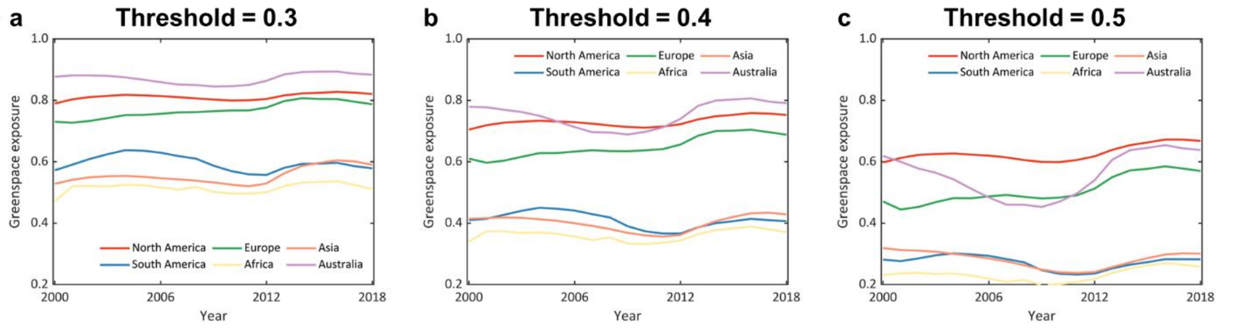

Supplementary Fig. 6. Sensitivity of threshold in the linear unmixing-based greenspace classification to the mean annual dynamics of real greenspace exposure (GE) for global 1028 cities from 2000-2018. **a.** Threshold = 0.3. **b.** Threshold = 0.4. **c.** Threshold = 0.5.

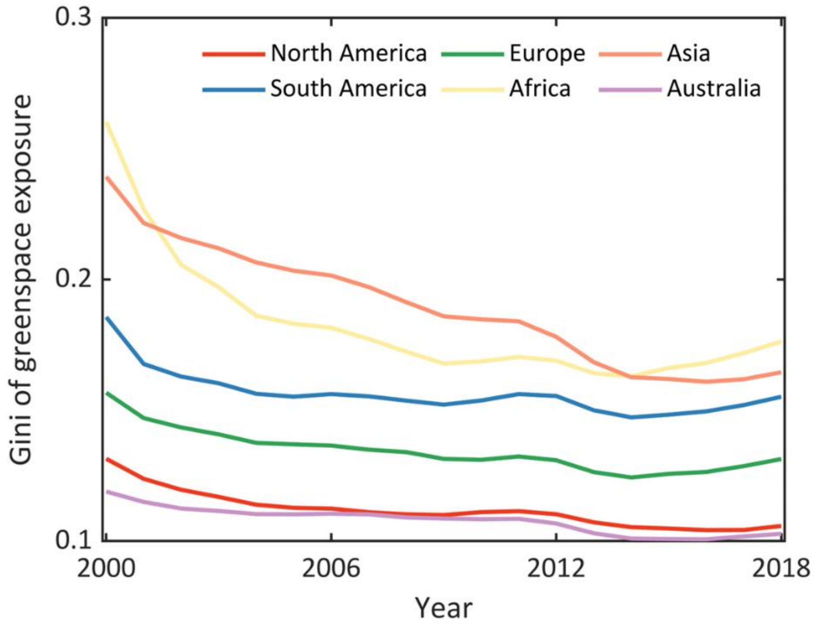

Supplementary Fig. 7. Continental-scale mean annual dynamics of greenspace exposure inequality measured by the Gini index for global 1028 cities from 2000-2018.

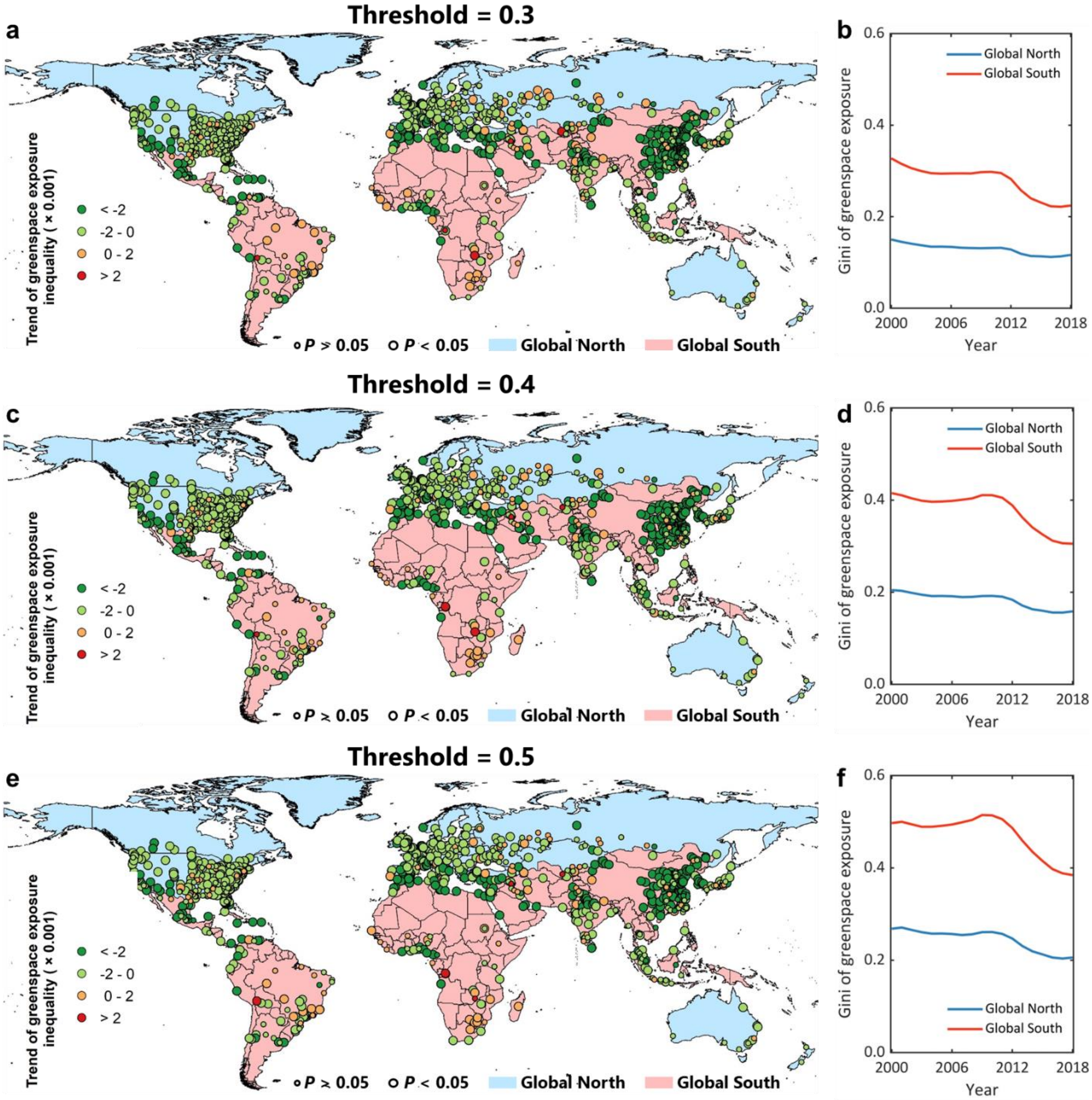

Supplementary Fig. 8. Sensitivity of threshold in the linear unmixing-based greenspace classification to the temporal changes of greenspace exposure inequality measured by the Gini index for global 1028 cities from 2000-2018. a and **b.** Threshold = 0.3. **c** and **d.** Threshold = 0.4. **e** and **f.** Threshold = 0.5. First column is the city-level temporal trend of Gini changes (**a, c,** and **e**). Second column is the mean annual Gini dynamics for Global North and Global South cities (**b, d,** and **f**). The non-parametric Theil–Sen slope estimator approach is used to determine the long-term trends of Gini. The non-parametric Mann-Kendall is used to evaluate the significance of these detected temporal trends. Large bubble sizes represent a statistically significant level of 0.05 (*p*-value <0.05) and small bubble sizes represent a non-significant trend with *p*-value >0.05. The administrative boundaries data is from the Global Administrative Areas (GADM) (https://gadm.org/).

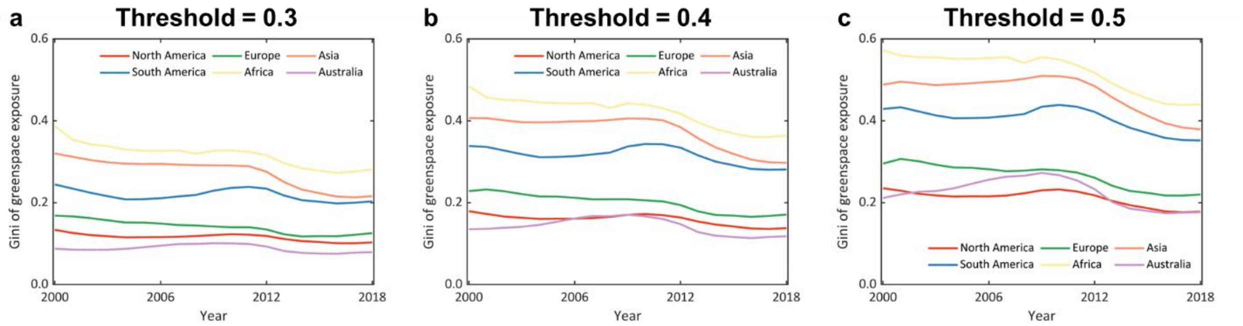

Supplementary Fig. 9. Sensitivity of threshold in the linear unmixing-based greenspace classification to the mean annual dynamics of greenspace exposure inequality measured by the Gini index for global 1028 cities from 2000-2018. **a.** Threshold = 0.3. **b.** Threshold = 0.4. **c.** Threshold = 0.5.

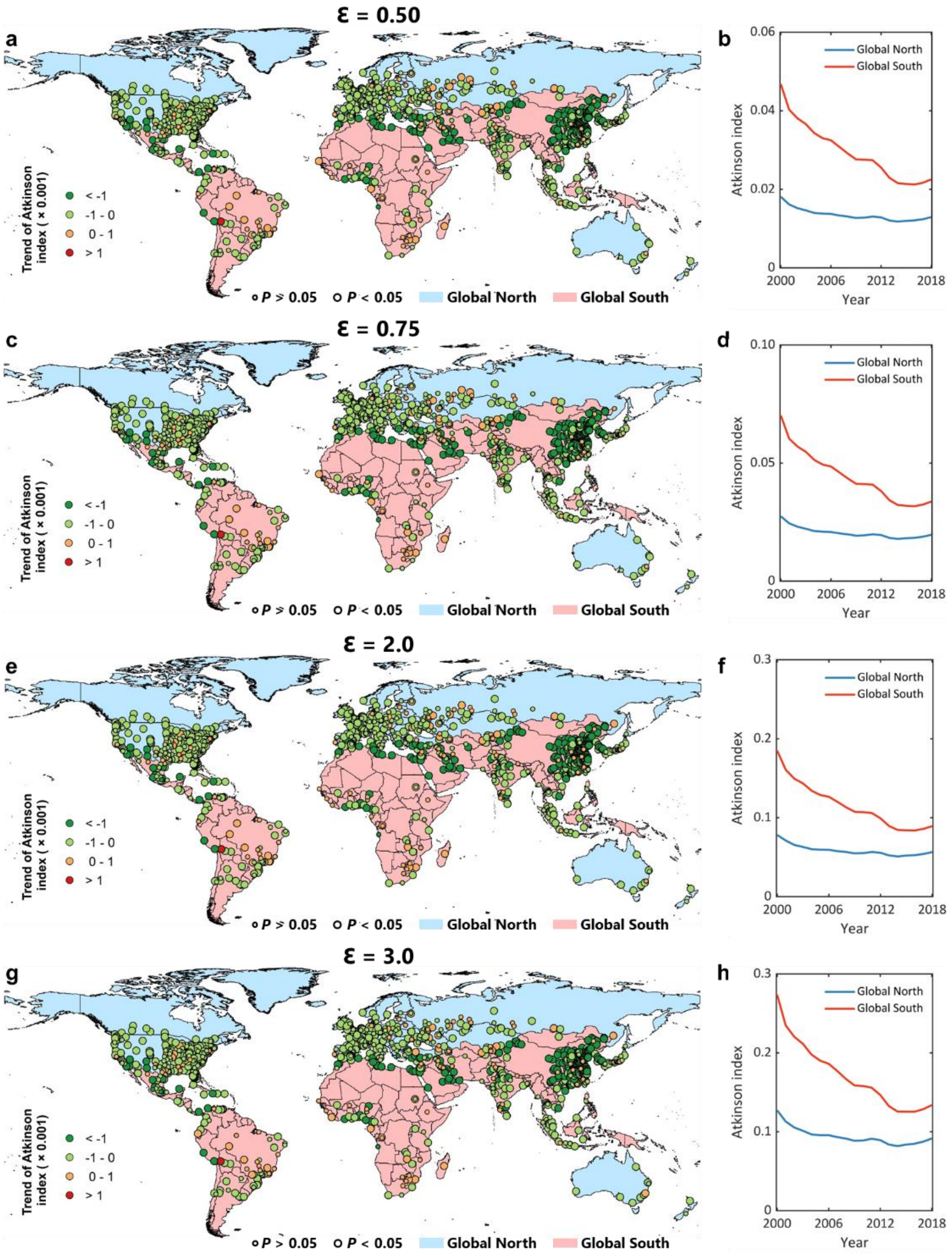

Supplementary Fig. 10. Temporal change of greenspace exposure inequality measured by the Atkinson index for global 1028 cities from 2000-2018. a and **b.** Inequality aversion parameter (ε) = 0.50. **c** and **d.** ε = 0.75. **e** and **f.** ε = 2.0. **g** and **h**. ε = 3.0. First column is the city-level temporal trend of Atkinson changes (**a, c, e,** and **g**). Second column is the mean annual Atkinson dynamics for Global North and Global South cities (**b, d, f,** and **h**). The non-parametric Theil–Sen slope estimator approach is used to determine the long-term trends of Atkinson. The non-parametric Mann-Kendall is used to evaluate the significance of these detected temporal trends. Large bubble sizes represent a statistically significant level of 0.05 (*p*-value <0.05) and small bubble sizes represent a non-significant trend with *p*-value >0.05. The administrative boundaries data is from the Global Administrative Areas (GADM) (https://gadm.org/).

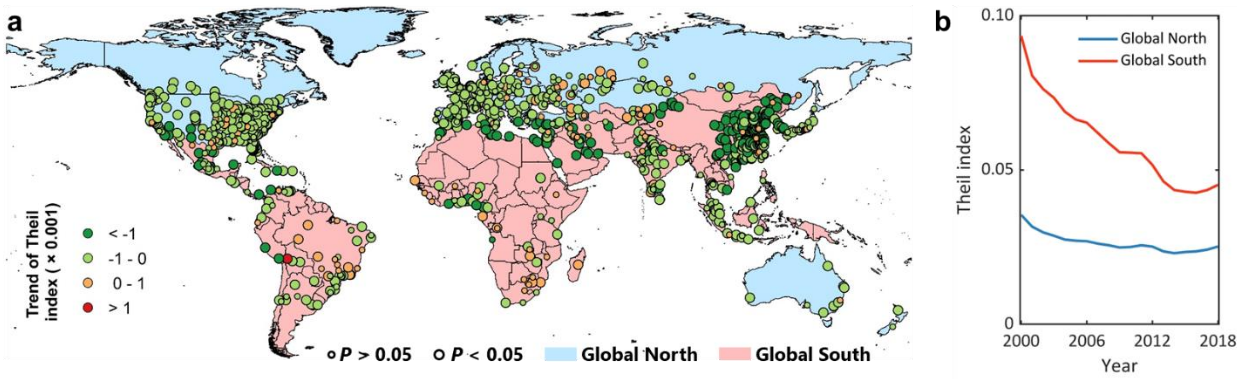

Supplementary Fig. 11. Temporal change of greenspace exposure inequality measured by the Theil index for global 1028 cities from 2000-2018. a. City-level temporal trend of Theil changes. **a.** Mean annual Theil dynamics for Global North and Global South cities. The non-parametric Theil–Sen slope estimator approach is used to determine the long-term trends of Theil. The non-parametric Mann-Kendall is used to evaluate the significance of these detected temporal trends. Large bubble sizes represent a statistically significant level of 0.05 (p-value <0.05) and small bubble sizes represent a non-significant trend with p-value >0.05. The administrative boundaries data is from the Global Administrative Areas (GADM) (https://gadm.org/).

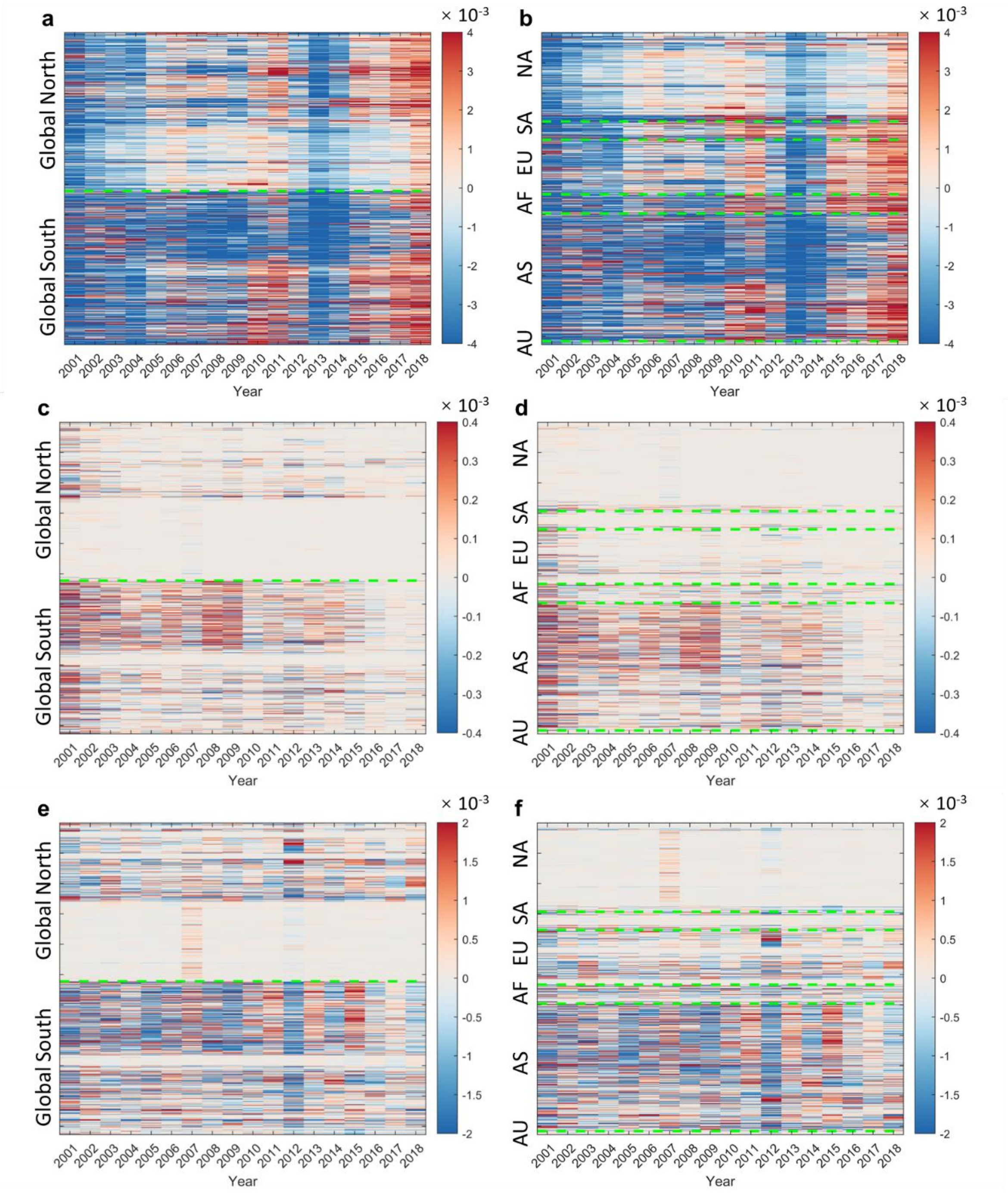

Supplementary Fig. 12. Individual and joint contributions of greenspace and population to the temporal change of the Gini index across global 1028 cities. a-b. Individual effect of greenspace. **c-d.** Individual effect of population. **e-f.** Joint effects of greenspace and population. Each line denotes the temporal change of one city. First column (**a, c,** and **e**) represents cities in the Global North and Global South region. Second column (**b, d,** and **f**) represents cities in different continents. AU: Australia. AS: Asia. AF: Africa. EU: Europe. SA: South America. NA: North America.

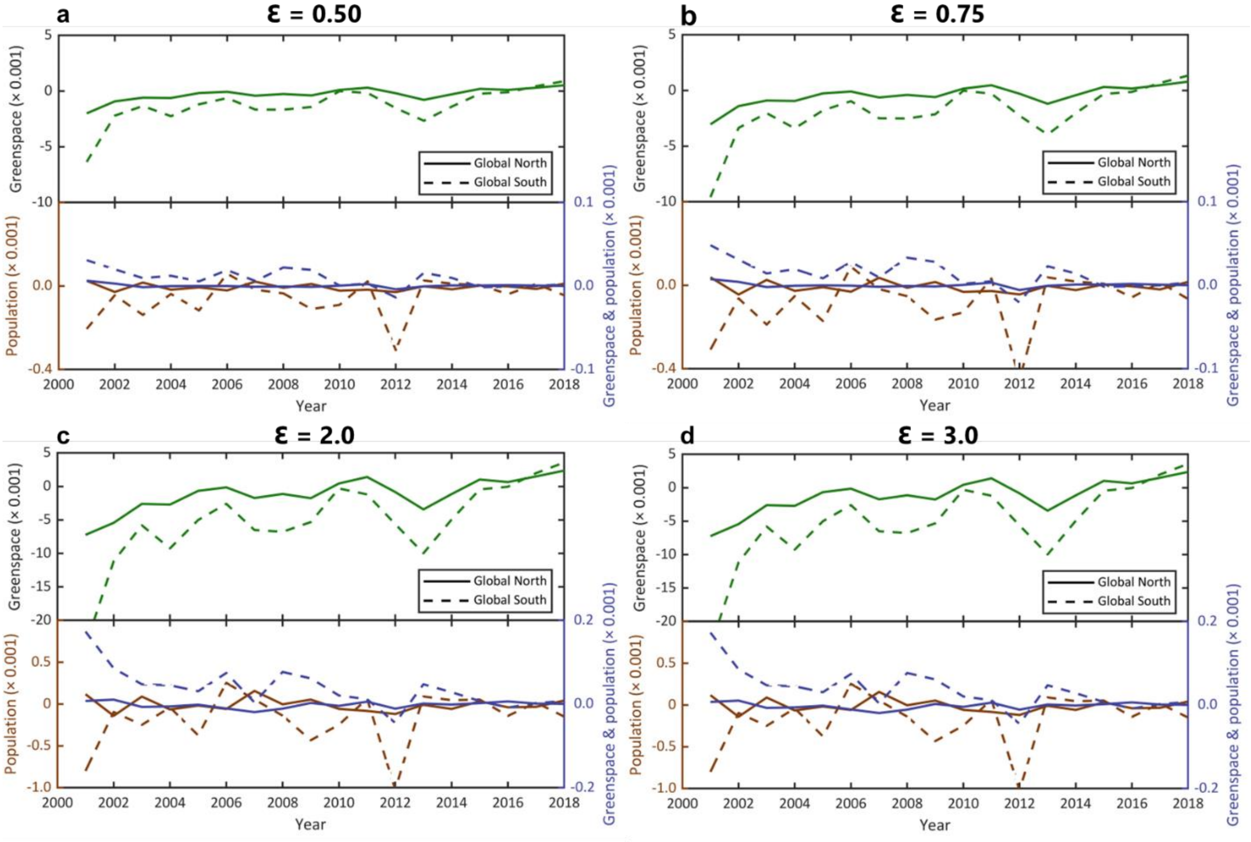

Supplementary Fig. 13. Attribution of drivers accounting for temporal change in greenspace exposure inequality measured by the Atkinson index. **a.** Inequality aversion parameter (ε) = 0.50. **b.** ε = 0.75. **c.** ε = 2.0. **d**. ε = 3.0.

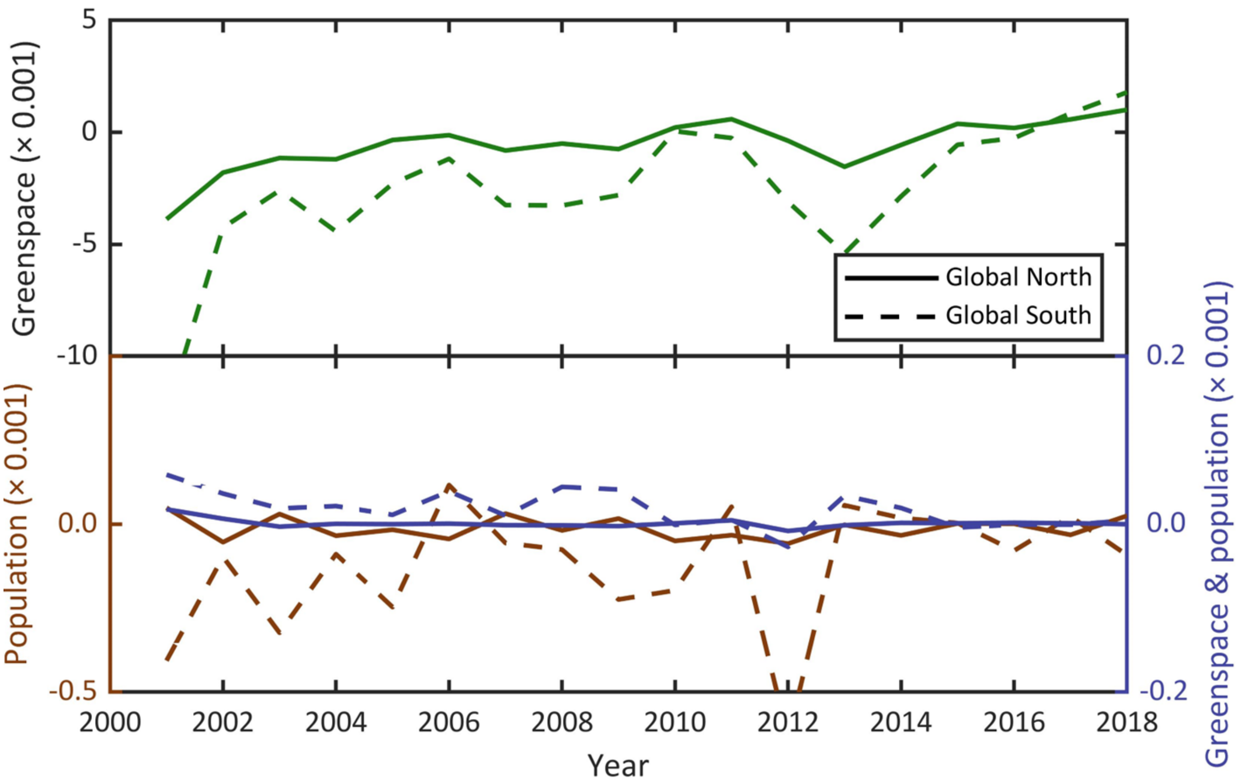

Supplementary Fig. 14. Attribution of drivers accounting for temporal change in greenspace exposure inequality measured by the Theil index.

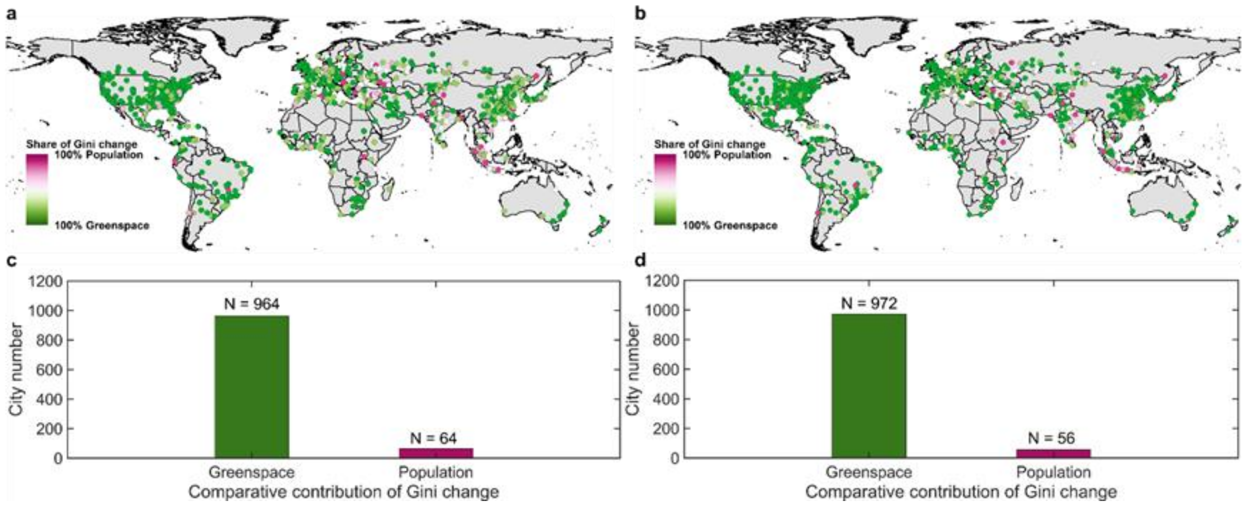

Supplementary Fig. 15. Comparative contributions of greenspace and population to the temporal change in the Gini index using the empirical approach proposed by Tuholske et al. (2020). a-b. Spatial patterns of population versus greenspace for the overall change of the Gini index using the comparative contribution (CC) metric: CC $=(|\beta_{\mathrm{pop}}| - |\beta_{\mathrm{green}}|) \div |\beta_{\mathrm{expo}}|$. **c-d.** City statistics from greenspace and population to the temporal change in greenspace exposure inequality. In **a** and **c**, the overall trend β_{expo} and the share of greenspace β_{green} are first calculated and then the share of population is quantified: $\beta_{pop} = \beta_{expo} - \beta_{green}$. In **b** and **d**, the overall trend β_{expo} and the share of population β_{pop} are first calculated and then the share of greenspace is quantified: $\beta_{green} = \beta_{expo} - \beta_{pop}$. The administrative boundaries data is from the Global Administrative Areas (GADM) (https://gadm.org/).

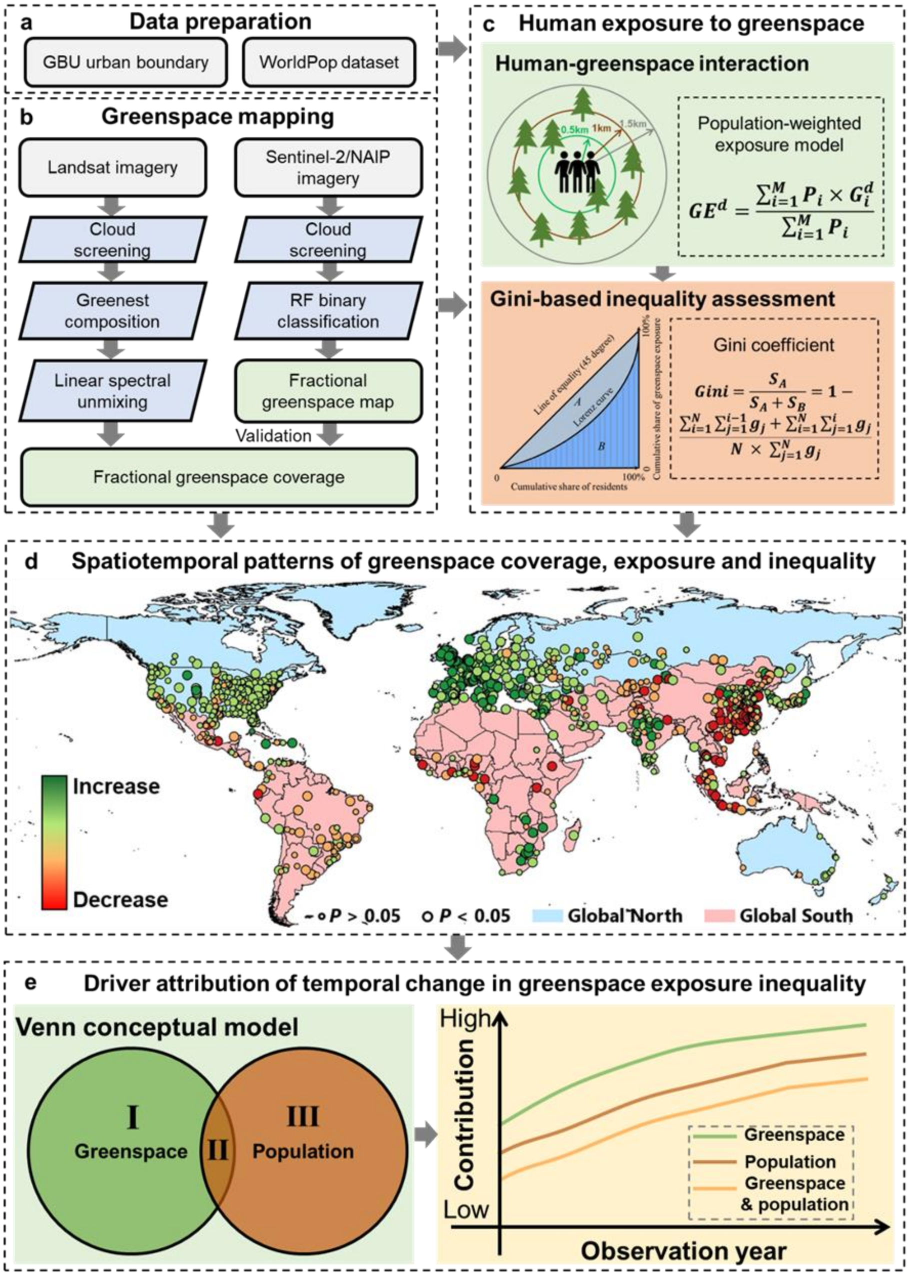

Supplementary Fig. 16. Flowchart of the research design in this study with five major steps. **a.** Data preparations of global city boundary and population datasets. **b.** Greenspace mapping from long-term Landsat satellite imagery with the linear spectral unmixing approach. **c.** human exposure to greenspace with population-weighted exposure model and inequality assessment. **d.** spatiotemporal analysis of physical greenspace coverage, human exposure, and inequality over global 1028 cities. **e.** Driver attribution of temporal change in greenspace exposure inequality over past two decades by accounting for individual greenspace and population effects, and their interaction effects. The administrative boundaries data is from the Global Administrative Areas (GADM) (https://gadm.org/).

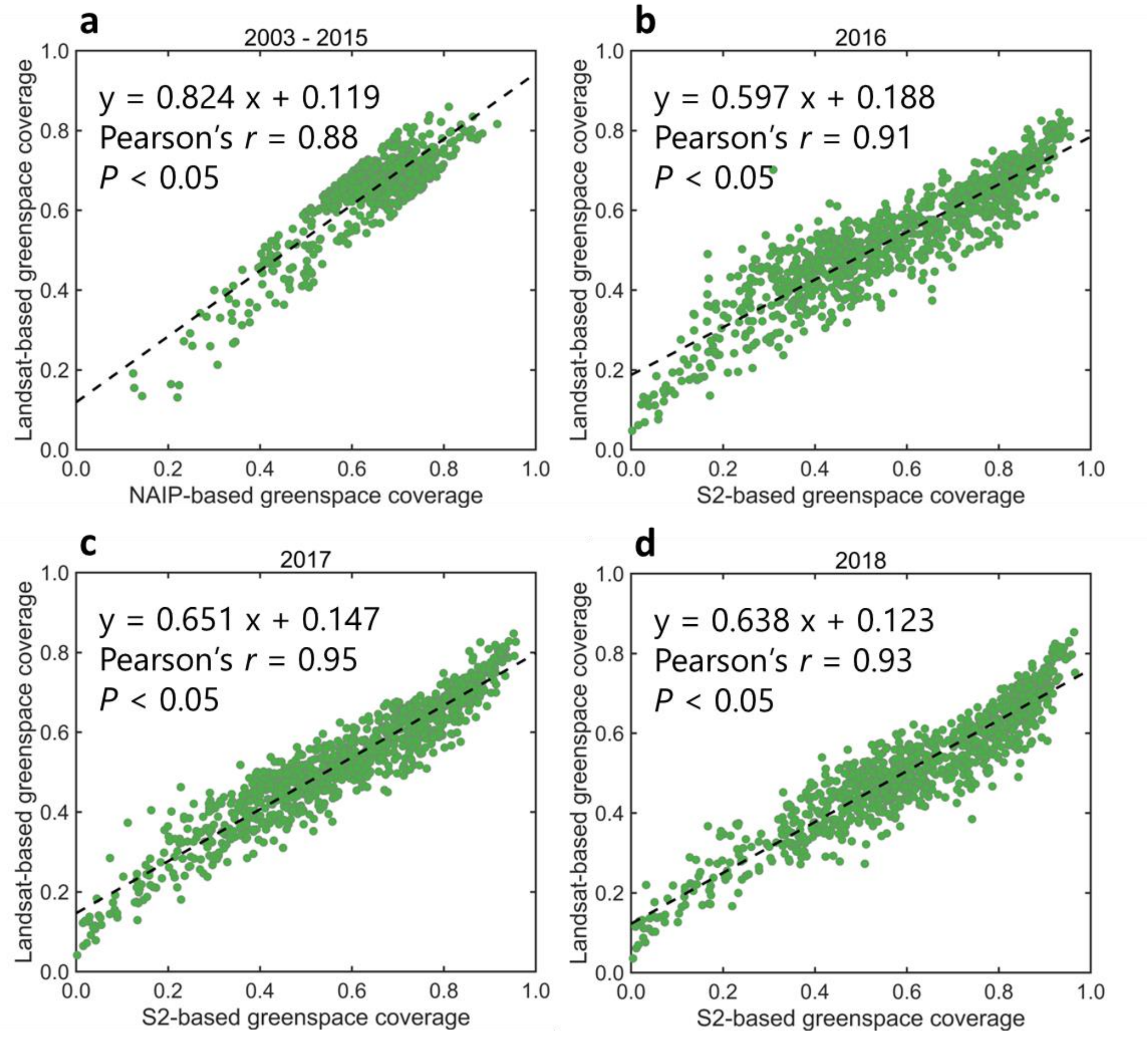

Supplementary Fig. 17. Comparison of physical greenspace coverage (GC) derived from 1-m NAIP and 10-m Sentinel-2 imageries (x-axis) and Landsat imagery (y-axis) across global 1028 cities. a. NAIP vs. Landsat in 2003-2015. **b-d.** Sentinel-2 vs. Landsat in 2016-2018. The GCs from NAIP and Sentinel-2 imageries are extracted from the vegetation and non-vegetation classification mapping with a random forest approach and then aggregated to city-level mean for comparison. Linear regression was used to measure their correlation with Pearson's r coefficient.

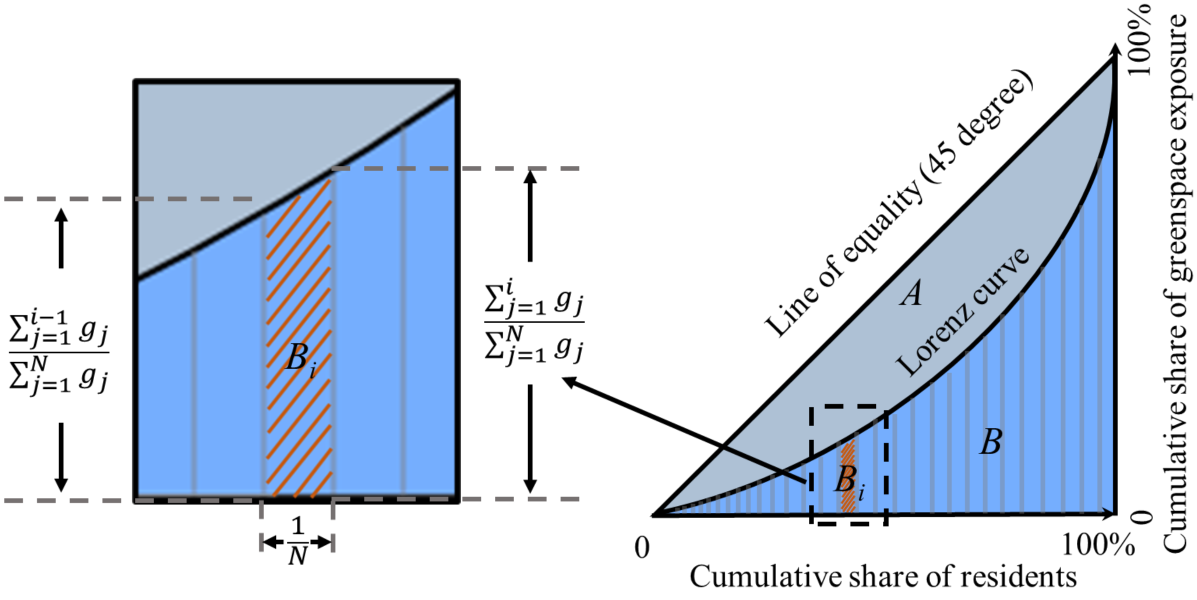

Supplementary Fig. 18. Illustrative diagram of Gini index for inequality assessments of greenspace exposure. The Gini index is defined as the ratio of the area that lies between the line of equality and the Lorenz curve (region *A*) over the total area under the line of equality (region *A* plus region *B*), where the Lorenz curve plots the proportion of the greenspace exposure (y-axis) that is cumulatively shared by the residents (x-axis). B_i indicates the contribution of *i*th residents to the accumulated greenspace exposure and is estimated by the trapezoid area as shown in the left panel, where g_i represents the greenspace that is exposed to *i*th resident, and *N* represents the resident number. Y-axis shows the cumulative share of greenspace exposure; X-axis shows the cumulative share of residents from lowest to highest greenspace exposure. This figure is revised from **Fig. A14** in Chen et al. 2022 [18].

Table S1. Statistics of temporal trends of city-level greenspace coverage, human exposure to greenspace, and greenspace exposure inequality across regions. The greenspace coverage is calculated by the spectral unmixing-based threshold classification approach, with a 0.3 threshold.

Region (# of cities)	Greenspace coverage (× 0.001 yr^{-1})	Greenspace exposure (× 0.001 yr^{-1})	Gini of greenspace exposure (× 0.001 yr^{-1})
Global North (522)	1.57 ± 3.57	2.41 ± 3.77	-1.80 ± 2.78
Global South (506)	-2.24 ± 5.89	1.60 ± 6.77	-5.16 ± 5.58
North America (293)	-0.58 ± 2.54	0.88 ± 2.56	-1.22 ± 2.52
South America (60)	-2.11 ± 3.69	-2.26 ± 4.38	-1.18 ± 2.92
Europe (180)	2.56 ± 3.26	4.32 ± 4.14	-2.77 ± 2.95
Africa (63)	-1.47 ± 4.76	0.16 ± 6.12	-4.20 ± 6.24
Asia (420)	-0.82 ± 6.75	2.74 ± 6.80	-5.60 ± 5.44
Australia/Oceania (12)	-0.12 ± 0.51	0.71 ± 1.10	-0.62 ± 0.47
Global (1028)	-0.20 ± 5.15	2.01 ± 5.47	-3.45 ± 4.70

Table S2. Statistics of temporal trends of city-level greenspace coverage, human exposure to greenspace, and greenspace exposure inequality across regions. The greenspace coverage is calculated by the spectral unmixing-based threshold classification approach, with a 0.4 threshold.

Region (# of cities)	Greenspace coverage ($\times$ 0.001 yr^{-1})	Greenspace exposure ($\times$ 0.001 yr^{-1})	Gini of greenspace exposure ($\times$ 0.001 yr^{-1})
Global North (522)	2.38 ± 4.19	3.46 ± 4.16	-2.65 ± 3.23
Global South (506)	-5.17 ± 7.44	-0.67 ± 6.71	-5.61 ± 5.94
North America (293)	-0.16 ± 3.05	1.90 ± 3.18	-1.82 ± 2.45
South America (60)	-2.47 ± 4.72	-2.14 ± 4.36	-2.44 ± 3.03
Europe (180)	4.42 ± 4.57	5.95 ± 4.37	-3.84 ± 3.28
Africa (63)	-1.36 ± 7.92	0.36 ± 6.98	-5.92 ± 7.12
Asia (420)	-4.08 ± 8.31	-0.19 ± 6.89	-5.85 ± 5.97
Australia/Oceania (12)	1.17 ± 1.09	2.26 ± 2.43	-1.67 ± 1.00
Global (1028)	-1.13 ± 7.01	1.43 ± 5.93	-4.11 ± 4.99

Table S3. Statistics of temporal trends of city-level greenspace coverage, human exposure to greenspace, and greenspace exposure inequality across regions. The greenspace coverage is calculated by the spectral unmixing-based threshold classification approach, with a 0.5 threshold.

Region (# of cities)	Greenspace coverage (× 0.001 yr^{-1})	Greenspace exposure (× 0.001 yr^{-1})	Gini of greenspace exposure (× 0.001 yr^{-1})
Global North (522)	3.55 ± 5.52	4.72 ± 4.66	-3.68 ± 3.67
Global South (506)	-6.74 ± 8.93	-1.64 ± 6.78	-5.82 ± 6.15
North America (293)	0.69 ± 3.78	3.20 ± 3.65	-2.66 ± 2.74
South America (60)	-2.15 ± 5.18	-1.17 ± 3.85	-3.76 ± 3.09
Europe (180)	6.92 ± 6.81	7.67 ± 5.01	-5.10 ± 3.75
Africa (63)	0.26 ± 8.25	1.68 ± 7.18	-7.42 ± 7.00
Asia (420)	-6.25 ± 9.50	-1.84 ± 6.80	-5.80 ± 6.31
Australia/Oceania (12)	4.10 ± 2.74	4.58 ± 3.69	-3.31 ± 2.08
Global (1028)	-1.23 ± 8.92	1.59 ± 6.61	-4.73 ± 5.16

Table S4. Statistics of temporal trends of greenspace exposure inequality measured by the Atkinson index across regions. Four inequality aversion parameters of 0.50, 0.75, 2.0, and 3.0 are used.

Region (# of cities)	Atkinson, $\varepsilon = 0.50$ (× 0.001 yr^{-1})	Atkinson, $\varepsilon = 0.75$ (× 0.001 yr^{-1})	Atkinson, $\varepsilon = 2.0$ (× 0.001 yr^{-1})	Atkinson, $\varepsilon = 3.0$ (× 0.001 yr^{-1})
Global North (522)	-0.20 ± 0.35	-0.31 ± 0.53	-0.84 ± 1.49	-1.35 ± 2.41
Global South (506)	-1.15 ± 1.43	-1.73 ± 2.09	-4.54 ± 5.08	-6.51 ± 7.15
North America (293)	-0.23 ± 0.66	-0.34 ± 0.95	-0.87 ± 2.15	-1.30 ± 3.06
South America (60)	-0.26 ± 0.78	-0.40 ± 1.14	-1.15 ± 3.05	-1.77 ± 4.20
Europe (180)	-0.26 ± 0.30	-0.39 ± 0.46	-1.07 ± 1.26	-1.78 ± 2.18
Africa (63)	-0.81 ± 1.53	-1.23 ± 2.24	-3.39 ± 5.75	-5.01 ± 8.29
Asia (420)	-1.21 ± 1.36	-1.81 ± 2.00	-4.76 ± 4.89	-6.81 ± 6.91
Australia/Oceania (12)	-0.14 ± 0.08	-0.22 ± 0.13	-0.68 ± 0.44	-1.23 ± 0.92
Global (1028)	-0.67 ± 1.14	-1.01 ± 1.67	-2.66 ± 4.15	-3.89 ± 5.89

Table S5. Statistics of temporal trends of greenspace exposure inequality measured by the Theil index across regions

Region (# of cities)	Theil (× 0.001 yr^{-1})
Global North (522)	-0.41 ± 0.68
Global South (506)	-2.29 ± 2.99
North America (293)	-0.47 ± 1.47
South America (60)	-0.52 ± 1.54
Europe (180)	-0.51 ± 0.60
Africa (63)	-1.63 ± 3.26
Asia (420)	-2.39 ± 2.81
Australia/Oceania (12)	-0.27 ± 0.15
Global (1028)	-1.33 ± 2.35

Table S6. The numbers of NAIP image and city used in this study for the accuracy validation of Landsat-derived greenspace coverage.

Year	Image number	City number
2003	668	20
2004	1302	45
2005	2231	74
2006	1976	69
2007	641	25
2008	677	23
2009	697	33
2010	2197	75
2011	1192	38
2012	3177	76
2013	1947	66
2014	1434	42
2015	1441	53

Supplementary references

1 Atkinson AB, Bourguignon F. Handbook of income distribution. Elsevier (2014).

2 Sitthiyot T, Holasut K. A simple method for measuring inequality. *Palgrave Communications* **6**, 1-9 (2020).

3 Chen B, Wu S, Song Y, Webster C, Xu B, Gong P. Contrasting inequality in human exposure to greenspace between cities of Global North and Global South. *Nature Communications* **13**, 1-9 (2022).

4 Song Y, et al. Observed inequality in urban greenspace exposure in China. *Environment International* **156**, 106778 (2021).
